# Interleukin-6, but not the interleukin-6 receptor plays a role in recovery from dextran sodium sulfate-induced colitis

**DOI:** 10.3892/ijmm.2014.1825

**Published:** 2014-06-27

**Authors:** JAN SOMMER, ERIKA ENGELOWSKI, PAUL BARAN, CHRISTOPH GARBERS, DOREEN M. FLOSS, JÜRGEN SCHELLER

**Affiliations:** 1 Institute of Biochemistry and Molecular Biology II, Medical Faculty, Heinrich-Heine-University, D-40225 Düsseldorf, Germany; 2 Institute of Biochemistry, Medical Faculty, Christian-Albrechts-University, D-24098 Kiel, Germany

**Keywords:** inflammation, ulcerative colitis, cytokine, dextran sodium sulfate, animal model

## Abstract

Interleukin *(IL)-6*-deficient, but not IL-6 receptor (*IL-6R*)-deficient mice present with a delayed skin wound healing phenotype. Since IL-6 solely signals via the IL-6R and glycoprotein 130 (gp130), *Il-6r*-deficient mice are expected to exhibit a similar phenotype as *Il-6*-deficient mice. However, p28 (IL-30) and ciliary neurotrophic factor (CNTF) have been identified as additional low-affinity ligands of the IL-6R/gp130/LIFR complex. IL-6 plays an inflammatory and regenerative role in inflammatory bowel disease (IBD). In the present study, we compared *Il-6r*-deficient mice with mice treated with neutralizing IL-6 monoclonal antibody (mAb) in a model of dextran sodium sulfate (DSS)-induced colitis. Our results, in agreement with those of previous reports, demonstrated that IL-6 mAbs slightly attenuated DSS-induced colitis during the regeneration phase. *Il-6r*-deficient mice and mice with tissue-specific deletion of the *Il-6r* in the myeloid cell lineage (LysMCre) with acute and chronic DSS-induced colitis were, however, indistinguishable from wild-type mice. Our data suggest that IL-6 and IL-6R have an additional role in colitis, apart from the IL-6/IL-6R classic and trans-signaling.

## Introduction

Autoimmune diseases comprise a variety of different malignancies, including at least two distinct forms of inflammatory bowel disease (IBD), namely Crohn’s disease (CD) and ulcerative colitis (UC) (1). In North America, UC has a prevalence of 150–300 cases per 100,000 inhabitants, while the incidence is between 2.5 to 20 cases per 100,000 inhabitants each year (2). Even though environmental factors in combination with genetic predisposition trigger the disease (3), the detailed pathophysiological mechanisms remain unclear.

The levels of interleukin (IL)-6 in sera positively correlate with disease severity in IBD (4). IL-6 is mainly secreted from lamina propria mononuclear cells and T cells in patients with CD (5,6). As previously demonstrated, in an acquired immunity-dependent T cell transfer colitis mouse model, IL-6 receptor (IL-6R) monoclonal antibodies (mAbs) prevented the development of colitis (7). The neutralizing IL-6R mAb, tocilizumab, represents a promising drug for CD (8). Importantly, IL-6 trans-signaling via IL-6/soluble IL-6 receptor (sIL-6R) complexes, but not classic signaling via IL-6/membrane bound IL-6 receptor (IL-6R) complexes has been shown to prevent the apoptosis of T cells and promote tissue damage (9). IL-6 trans-signaling also promotes the development of spontaneous ileitis in SAMP1/Yit mice (10). On the other hand, IL-6 stimulates the survival and proliferation of intestinal epithelial cells. The abrogation of regenerative pathways in the intestine may explain why *Il-6*-deficient mice present with a widespread damage of the colonic mucosa in a non-T cell-dependent innate immunity-dependent azoxymethane (AOM)-dextran sodium sulfate (DSS)-induced colitis model (11), which is accompanied with decreased tumor incidence (11). However, an earlier study reported reduced colitis and mortality after DSS administration in *Il-6*-deficient mice compared to wild-type (wt) mice (12).

*Il-6r*-deficient mice are expected to exhibit a similar phenotype as *Il-6*-deficient mice, since IL-6 solely signals via the IL-6R and glycoprotein 130 (gp130). This view has been challenged with the identification of ciliary neurotrophic factor (CNTF) as an additional low-affinity ligand of the IL-6R/gp130/LIFR complex (13), and p28 (IL-30) as a ligand of the IL-6R/gp130 complex (14,15). It is interesting to note that, in contrast to *Il-6*-deficient mice, *Il-6r*-deficient mice do not exhibit a delay in skin wound closure (16), pointing to additional roles of IL-6 or the IL-6R apart from the IL-6/(s)IL-6R classic and trans-signaling.

In the present study, we analyzed IL-6 mAb-treated and *Il-6r*-deficient mice in a model of DSS-induced colitis. DSS causes the destruction of intestinal epithelial cells accompanied by subsequent intestinal inflammation and regeneration (17,18).

In agreement with a previous report (12) using *Il-6r*-deficient mice, our results demonstrated that neutralizing IL-6 mAbs slightly improved DSS-induced colitis. *Il-6r*-deficient mice and mice with tissue-specific deletion of the *Il-6r* in the myeloid cell lineage (LysMCre) with acute and chronic DSS-induced colitis were, however, indistinguishable from wt mice.

## Materials and methods

### Ethics statement

All mouse experiments were performed according to the requirements of Landesamt für Natur, Umwelt und Verbraucherschutz Nordrhein-Westfalen (LANUV NRW) with the following approval number: 84-02.04.2011.A146.

### Animals

Specific pathogen-free *Il-6r^fl/fl^* and Cre-recombinase-expressing mice, as well as their offspring were obtained from the animal facility of the University of Düsseldorf (Düsseldorf, Germany). All mice had a C57BL/6 background. The mice were fed a standard laboratory diet and were provided with autoclaved tap water *ad libitum*. They were kept in an air-conditioned room with a controlled temperature (20–24°C), humidity (45–65%) and day/night cycle (12 h light, 12 h dark). The mice were allowed to acclimatize for 1 week prior to entering the study. Each group in our model of DSS-induced colitis contained 4–16 animals. The animals were monitored daily for changes in behaviour and body weight loss. Animals presenting severe signs of suffering (weight loss of >20%, prostration and tremors) were euthanized.

### DSS-induced colitis

Acute DSS colitis was induced by the administration of 1.5–3% DSS in the drinking water for 5 days followed by 5 days of autoclaved tap water. The DSS-containing water was changed on day 3. On day 10, the mice were anesthetized using xylazin (10 mg/kg body weight) and ketamin (100 mg/kg body weight). The mice were coloscopied (endoscopy system AIDA control; Storz, Tuttlingen, Germany) and images of the colon were acquired. Subsequently, the mice were dissected (ventral), blood samples were acquired by cardiac puncture and the colon was prepared in one section, washed in PBS and continued to be processed as described in the sections ‘Immunohistochemistry’ and ‘Colon organ culture’.

### Determination of clinical score

The clinical score was assessed without taking the genotype of the mice into consideration and determined taking into account the weight loss compared to day 0, stool consistency and blood in the stool, as previously described (19). No weight loss or up to 5% weight loss was registered as 0 points, weight loss of >5% up to 10% as 1 point, >10% up to 15% 2 points, >15% up to 20% 3 points, and >20% 4 points. For stool consistency, 0 points were assigned for well-formed pellets, 1 point for pasty and semi-formed stools and 2 points for liquid stools. To assess bleeding, 0 points were assigned for no blood by using the haemoccult test (HemoCare, Care Diagnostica Laborreagenzien GmbH, Voerde, Germany), 1 point for a positive haemoccult test and 2 points for visible bleeding.

### Administration of neutralizing IL-6 mAbs

Male *Il-6r^fl/fl^* mice were intraperitoneally injected with 250 μg rat IL-6 mAbs (MP5-20F3) 1 h prior to replacing the tap-water with 1.5% DSS-containing autoclaved tap water (day 0). The injection was repeated on days 2, 4, 6 and 8.

### Generation and genetic analysis of transgenic mice

The *Il-6r^fl/fl^* mice were crossed with mice expressing Cre-recombinase under the control of the lysozyme M (LysM) promoter (20) or a human cytomegalovirus (CMV) minimal promoter, as previously described (21). Progeny LysMCre^+/−^/*Il-6r^fl/+^* mice were further bred with LysMCre^−/−^/*Il-6r^fl/fl^* mice. The resulting offspring was kept, breeding LysMCre^+/−^/*Il-6r^fl/fl^* with LysMCre^−/−^/*Il-6r^fl/fl^* mice. The littermates were used for the experiments. Human CMVCre^+/−^/*Il-6r*^−^*^/+^* mice were bred with *Il-6r^fl/fl^* mice. From the resulting offspring, the *Il-6r^fl/fl^* or *Il-6r*^−^*^/^*^−^ mice were used for the experiments.

DNA from the tail clippings was isolated using the DirectPCR-Tail kit with proteinase K (Peqlab Biotechnologie GmbH, Erlangen, Germany) following the instructions of the manufacturer.

Primers for the wild-type *Il-6r* allele (5′-GGTCACGGG CACTCCTTGGATAGGTACC-3′ and 5′-CCCAGTGAGCT CCACCATCAAA-3′), the floxed *Il-6r* allele (5′-GGTCACGG GCACTCCTTGGATAGGTACC-3′ and 5′-CCCAGTGAGC TCCACCATCAAA-3′), the excised *Il-6r* allele (5′-GGGTAG GCCCTGCTACCATGAAG-3′ and 5′-CCCAGTGAGCTCC ACCATCAAA-3′) and human *CMVCre* (5′-ACGACCAAGT GACAGCAATG-3′ and 5′-TCGACCAGTTTAGTTACCC-3′) were used in PCR analysis. *LysMCre* mice were genotyped as described in the JAX mouse database (The Jackson Laboratory, Bar Harbor, ME, USA).

### Preparation of cells

Spleen and femoral bones were isolated from the LysMCre^+/−^*/Il-6r^fl/fl^* and *Il-6r^fl/fl^* mice. Spleen and bone marrow cells were passed through a nylon mesh into a 50-ml tube containing 10 ml PBS. The cell suspension was centrifuged at 290 × g for 5 min at room temperature. The pellet was resuspended with erythrocyte lysing buffer (150 mM NH_4_Cl, 10 mM KHCO_3_ and 100 nM EDTA), incubated for 5 min at room temperature and centrifuged again at 290 × g for 5 min. These steps were repeated until the supernatant lost its red colour completely. The pellet was finally resuspended in 100 μl flow cytometry buffer [1% (w/v) BSA in PBS].

### Flow cytometry

To analyze the cell-surface expression of IL-6R, cells (prepared as described in ‘Preparation of cells’) were incubated in 100 μl flow cytometry buffer containing 0.5 μl CD16/CD32 mAbs (Mouse BD Fc Block, 2.4G2; BD Pharmigen, San Jose, CA, USA) for 5 min on ice. Diluted IL-6R-PE mAbs (1 μl) (D7715A7; BioLegend, San Diego, CA, USA) and 3 μl diluted CD11b-APC mAbs (M1/70; BD Pharmigen) were added and the samples were incubated on ice for a further 30 min in the dark. After a single washing step in the flow cytometry buffer, the cells were resuspended in the flow cytometry buffer and were analyzed by flow cytometry (FACSCanto II and FACSDiva software; BD Biosciences, Heidelberg, Germany).

### Chronic colitis

Chronic colitis was induced in the LysMCre^+/−^/*Il-6r^fl/fl^* mice and their littermate controls. Colitis was induced as described above. DSS (1.5%) was administered in the drinking water for 5 days, followed by 7 days of autoclaved tap water. After the first cycle, 1.0% DSS was used for the second cycle and performed like the first; for the third cycle, we used 1.25% DSS and for the final cycle 1.5% DSS. On days 47 and 48, the mice were anesthetized using xylazin (10 mg/kg body weight) and ketamin (100 mg/kg body weight). The subsequent procedure was performed as described in ‘DSS-induced colitis’.

### Serum preparation

Serum was generated by allowing blood to clot at room temperature for 30 min and subsequent centrifugation at 2500 × g for 40 min.

### FITC-dextran measurement

Intestinal permeability was assessed by the administration of non-metabolizable FITC-dextran 4000 (TdB Consultancy, Uppsala, Sweden) (0.6 g/kg body weight) by gavage 4 h before sacrifice. Whole blood was obtained by cardiac puncture. Dilutions of FITC-dextran 4000 in the DMEM high-glucose culture medium (Life Technologies-Gibco, Darmstadt, Germany) were used as a standard curve and absorption of 50 μl serum diluted in DMEM high-glucose culture medium was measured in duplicate on the Tecan Infinite 200 Pro fluorometer (excitation wavelength, 488 nm; emission wavelength, 519 nm; software Tecan i-control; Tecan Deutschland GmbH, Crailsheim, Germany). The concentration of FITC-dextran 4000 was determined and standard errors were calculated.

### Colon organ culture

A segment of the distal colon was removed, cut longitudinally and washed in PBS. A segment of approximately 0.5 cm^2^ was incubated in a 24-well plate in DMEM high-glucose culture medium supplemented with penicillin (60 mg/l; Life Technologies-Gibco) and streptomycin (100 mg/l; Gibco) at 37°C with 5% CO_2_ in a water-saturated atmosphere for 24 h. The cells were removed from the medium and were centrifuged at 20,000 × g for 2 min. The supernatants were stored at −20°C before proceeding with ELISA procedure for quantification.

### ELISA quantification of IL-6 and IL-6R

ELISA for IL-6 (mouse interleukin-6 DuoSet; R&D Systems, Minneapolis, MN, USA) and IL-6R (mouse interleukin-6 sR DuoSet; R&D Systems) was performed following the manufacturer’s instructions. The peroxidase reaction was terminated by the addition of 50 μl 1.8 N H_2_SO_4_. The absorbance was determined using the Tecan Infinite 200 Pro fluorometer (absorption wavelength, 450 nm; software Tecan i-control).

### Immunohistochemistry

The remaining stool was removed from the colon by flushing with PBS. The colon was opened longitudinally, washed with PBS, cut in the middle longitudinally and one part was formed as a swiss-roll, as previously described (22). The colonic swiss-roll was incubated overnight in 4% (w/v) paraformaldehyde (PFA) in PBS. The liquid was replaced with pure PBS and the sections were incubated for another night. Tissues were dehydrated using Tissue-Tek^®^ VIP™ 5Jr (Sakura Finetek Germany GmbH, Staufen, Germany) in an ascending ethanol series followed by UltraClear (J.T. Baker Inc., Phillipsburg, NJ, USA) and paraffin. The samples were embedded in paraffin and dissected into 3-μm-thick slices using a microtome (Leica RM 2135). The sections were mounted on microscope slides, deparaffinized and rehydrated using a descending xylene and ethanol series. Tissue sections were stained using Mayer’s hematoxylin solution (Merck, Darmstadt, Germany) and differentiated in pure ethanol containing 2 g HCl per 100 ml. The tissue sections were washed and further stained using a 1.5% aqueous eosin Y solution (Formafix Global Technologies, Schleswig-Holstein, Germany). The stained sections were dehydrated using an ascending ethanol and xylene series, embedded with a cover slip and subsequently analyzed under a microscope (fluorescence lifetime imaging microscope BZ-9000; Keyence Corp., Osaka, Japan).

### RNA and cDNA

RNA was extracted from the tissues using the PeqGOLD Total RNA kit S-Line (Peqlab Biotechnologie GMBH, Erlangen, Germany) following the manufacturer’s instructions. RNA (5 μg) was used for cDNA synthesis using RevertAid reverse transcriptase (Thermo Fisher Scientific, Waltham, MA, USA) and oligo(dT) primers.

### qPCR

Using SYBR-Green PCR Master Mix (Applied Biosystems, Carlsbad, CA, USA), the qPCR reaction was performed in triplicate using 25 ng of the cDNA as a template. The fluorescence detection and measurements were taken using the Applied Biosystems thermal cycler. The relative expression levels of transforming growth factor (*Tgf)-β*, *Il-1*, tumor necrosis factor (*Tnf)-α*, *Kc* and *Vα14* for the DSS-challenged LysMCre^+/−^/*Il-6r^fl/fl^* mice and their littermate controls, as well as for the unchallenged LysMCre^+/−^/*Il-6r^fl/fl^* mice, was calculated after normalization with their unchallenged littermate controls. Primers were used as previously described (23). *Gapdh* was used as a housekeeping control. The resulting values were then averaged and plotted as a bar plot. Standard errors are presented in Table I.

### Statistical analysis

Data are expressed as the mean values ± standard deviation. Statistical analysis was performed using a Student’s t-test test and a p-value <0.05 was considered statistically significant.

## Results

### Neutralizing IL-6 mAbs slightly, although significantly attenuate DSS-induced colitis

**Figure 1:**
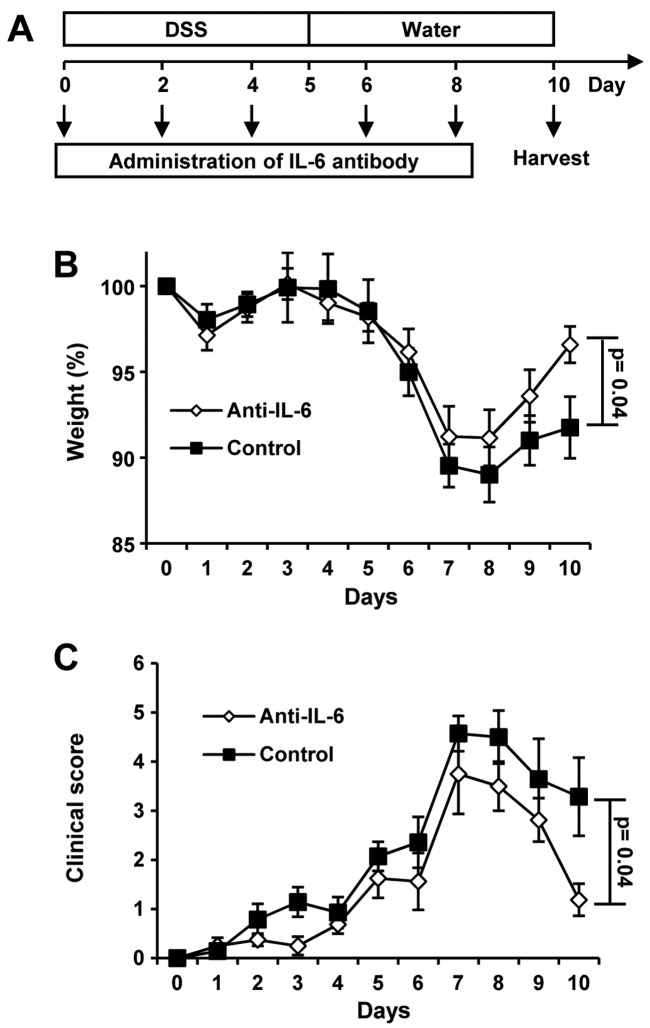
Neutralizing interleukin (IL)-6 in *Il-6r^fl/fl^* mice reduces weight loss and improves the clinical score in acute dextran sodium sulfate (DSS)-induced colitis. (A) Schematic overview of acute DSS-induced colitis as described in Materials and methods. The first injection of neutralizing IL-6 antibody or physiological salt solution was administered at the beginning of colitis (day 0). On days 2, 4, 6 and 8, the injections were repeated. On day 5, DSS-containing water was removed and replaced by autoclaved tap water for another 5 days. On day 10, the mice were anesthetized and analyzed as described in Materials and methods. (B) Weight curve during acute DSS-induced colitis for *Il-6r^fl/fl^* mice injected with physiological salt solution (control) and *Il-6r^fl/fl^* mice injected with neutralizing IL-6 antibody (anti-IL-6). Standard errors were calculated and indicated. Results of the Student’s t-test on day 10 are shown. (C) Clinical score during acute DSS-induced colitis for *Il-6r^fl/fl^* mice injected with physiological salt solution (control) and *Il-6r^fl/fl^* mice injected with neutralizing IL-6 antibody (anti-IL-6). Standard errors were calculated and are shown. Results of Student’s t-test on day 10 are shown.

For DSS-induced colitis, male mice were administered with 1.5% DSS in their drinking water, followed by 5 days of drinking water without DSS. One group received neutralizing IL-6 mAbs every other day (Fig. 1A).

We observed no weight loss in the mice until days 4 to 5. Maximal weight loss was monitored between days 6 to 7. After day 8, the mice began to gain weight during the recovery phase. Significant differences between the 2 groups were apparent only on day 10 (Fig. 1B). Statistically significant differences in the clinical scores between the 2 groups were also only detected on day 10 (Fig. 1C). Of note, the neutralization of IL-6 by mAbs promoted weight gain and improved the clinical score on day 10 as compared to the untreated wt mice. The body weight on day 10 was 92% for the control mice and 97% for the IL-6 mAb-treated mice. The clinical score was 3 for the control group and 1 for the IL-6 mAb-treated group. In agreement with the results presented in the study by Naito *et al* (12), our results revealed that IL-6 plays a detrimental role in DSS-induced colitis.

### Generation of global and tissue-specific IL-6R-deficient mice

**Figure 2:**
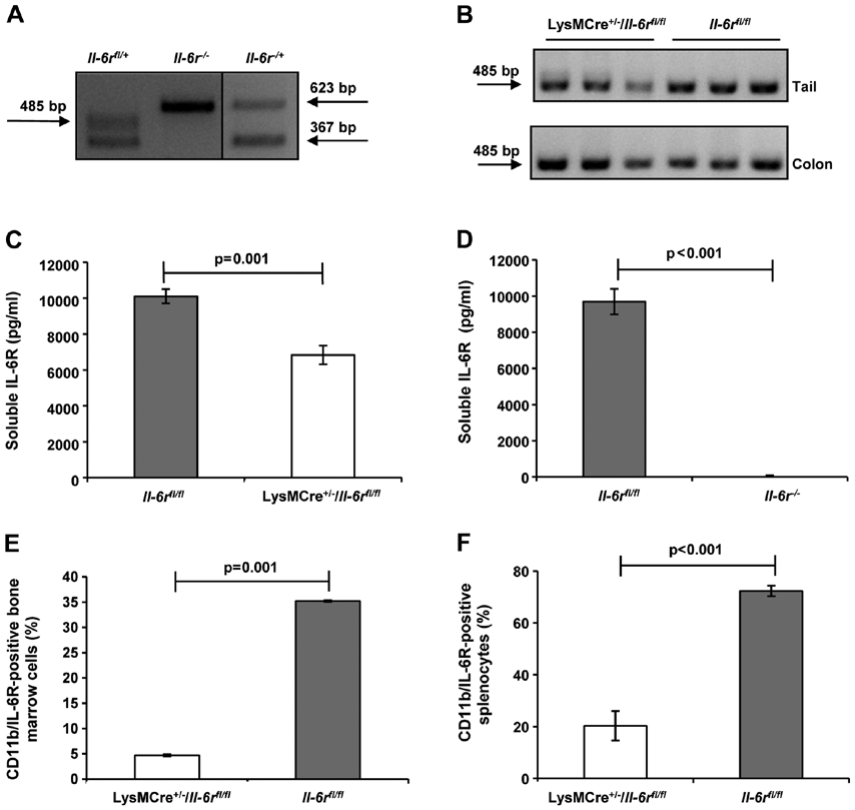
*Il-6r*^−^*^/^*^−^ mice do not express IL-6R and LysMCre^+/−^/*Il-6r^fl/fl^* mice do not express *Il-6r* in monocytes. (A) PCR of tail samples of *Il-6r^fl/+^*, *Il-6r*^−^*^/^*^−^ and *Il-6r*^−^*^/+^* mice. DNA was isolated and genotyped as described in Materials and methods. In the figure, 367 bp indicates the size of the wild-type allele, 485 bp indicates the size of the *Il-6r^fl/fl^* allele and 623 bp indicates the *Il-6r* recombination. (B) PCR of tail and colon samples of LysMCre^+/−^/*Il-6r^fl/fl^* and *Il-6r^fl/fl^* mice. DNA was isolated and genotyped as described in Materials and methods. In the figure, 485 bp indicates the size of the *Il-6r^fl/fl^* allele. (C) IL-6R ELISA for serum samples of unchallenged LysMCre^+/−^/*Il-6r^fl/fl^* mice and their littermate controls (*Il-6r^fl/fl^*). ELISA was performed as described in Materials and methods. Results of Student’s t-test are indicated. Standard errors were calculated and are shown. (D) IL-6R ELISA for serum samples of *Il-6r^fl/fl^* and *Il-6r*^−^*^/^*^−^ mice. ELISA was performed as described in Materials and methods. Results of Student’s t-test are indicated. Standard errors were calculated and are shown. (E) Flow cytometric analysis of bone marrow cells from LysMCre^+/−^/*Il-6r^fl/fl^* mice and their littermate controls (*Il-6r^fl/fl^*) stained for IL-6R and CD11b. Flow cytometric analysis was performed as described in Materials and methods. Results of Student’s t-test are indicated. Standard errors were calculated and are shown. (F) Flow cytometric analysis of splenocytes from LysMCre^+/−^/*Il-6r^fl/fl^* mice and their littermate controls (*Il-6r^fl/fl^*) stained for IL-6R and CD11b. Flow cytometric analysis was performed as described in Materials and methods. Standard errors were calculated and are shown. Results of Student’s t-test are indicated.

Conditional *Il-6r^fl/fl^* mice have been used in previous studies (16). In this study, *Il-6r^fl/fl^* mice were crossed with *CMVCre* and *LysMCre* transgenic mice, as previously described (20,21). Germline recombination of the *Il-6r* allele was achieved using *CMVCre*. The deletion of the *Il-6r* allele in the myeloid cell lineage, including monocytes, mature macrophages and granulocytes, was accomplished after the crossing of *Il-6r^fl/fl^* with *LysMCre* mice. Germline recombination was verified by PCR analysis of tail DNA from *Il-6r*^−^*^/^*^−^ mice (Fig. 2A). Unspecific recombination for LysMCre^+/−^/*Il-6r^fl/fl^* mice was excluded by PCR analysis of the colon and tail samples (Fig. 2B).

LysMCre^+/−^/*Il-6r^fl/fl^* mice have been previously described and present with approximately 60% reduced serum sIL-6R levels (16). In this study, our LysMCre^+/−^/*Il-6r^fl/fl^* mice had approximately 30% reduced serum levels of sIL-6R (Fig. 2C), even though our LysMCre^+/−^/*Il-6r^fl/fl^* mice showed a good recombination of the *Il-6r* allele. sIL-6R was not detectable in the *Il-6r*^−^*^/^*^−^ mice (Fig. 2D).

Subsequently, we analyzed the IL-6R cell surface expression on monocytes of LysMCre^+/−^/*Il-6r^fl/fl^* mice by flow cytometry. Bone marrow cells and splenocytes were stained for IL-6R and the monocyte marker, CD11b. Only 5% of the CD11b-positive bone marrow cells from the LysMCre^+/−^/*Il-6r^fl/fl^* mice, but approximately 35% of their littermate controls expressed the IL-6R (Fig. 2E). Only 20% of the CD11b-positive splenocytes from the LysMCre^+/−^/*Il-6r^fl/fl^* mice, but approximately 72% of their littermate controls expressed the IL-6R (Fig. 2F). Thus, it can be concluded from these experiments that the recombination of the IL-6R allele in the 2 transgenic mouse strains, *Il-6r*^−^*^/^*^−^ and LysMCre^+/−^/*Il-6r^fl/fl^*, was successful.

### Il-6r^−/−^ mice show no difference in susceptibility to acute DSS-induced colitis compared to Il-6r^fl/fl^ control mice

**Figure 3:**
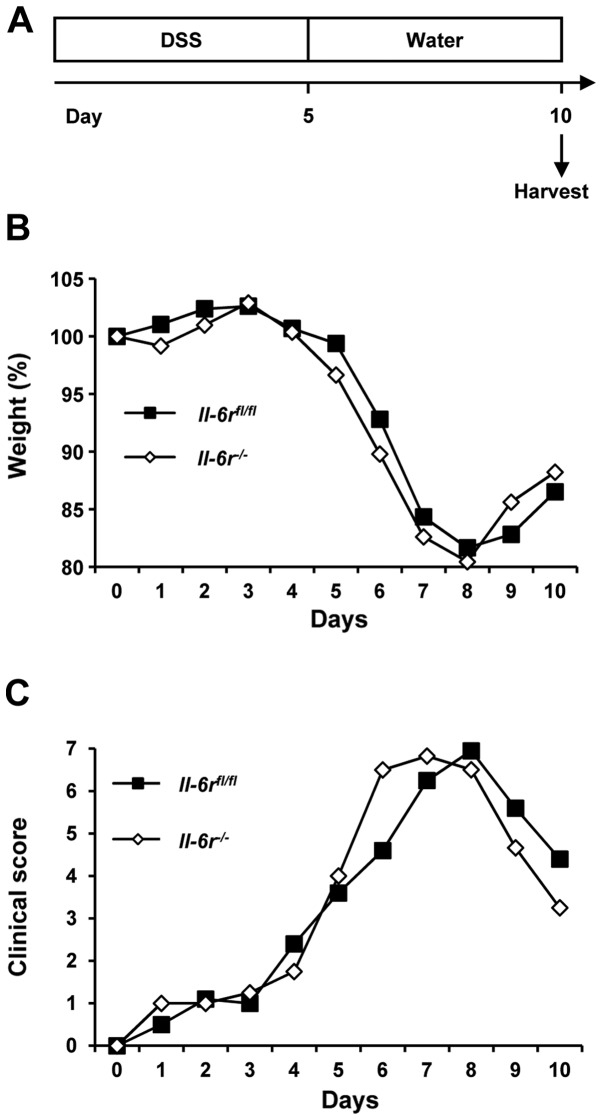
No difference was observed in weight loss and clinical score in acute dextran sodium sulfate (DSS)-induced colitis between *Il-6r^fl/fl^* and *Il-6r*^−^*^/^*^−^ mice. (A) Schematic overview of our experimental procedure of acute DSS-induced colitis. On day 5, DSS-containing water was removed and replaced by autoclaved tap water for another 5 days. On day 10, the mice were anesthetized and analyzed as described in Materials and methods. (B) Weight curve during acute DSS-induced colitis for *Il-6r^fl/fl^* and *Il-6r*^−^*^/^*^−^ mice. (C) Clinical score during acute DSS-induced colitis for *Il-6r^fl/fl^* and *Il-6r*^−^*^/^*^−^ mice.

After proving the successful recombination for the *Il-6r* allele, we created a mouse model of acute DSS-induced colitis using female *Il-6r^fl/fl^* and *Il-6r*^−^*^/^*^−^ mice (Fig. 3A). There was no weight loss in the mice until day 4. On day 5, the weight began to drop below 100% of the weight at the beginning of the experiment. The greatest weight loss occurred from day 5 to day 7. On day 8, the weight curves reached their minimum in both groups and began to rise again until the end of the experiment. No significant difference in weight loss was observed between the *Il-6r^fl/fl^* and *Il-6r*^−^*^/^*^−^ mice during the entire experimental period (Fig. 3B). The clinical score began to markedly increase on day 4, reaching maximum values for the *Il-6r*^−^*^/^*^−^ mice on day 7 and for the *Il-6r^fl/fl^* mice on day 8; thereafter, the clinical score began to decrease until the end of the experiment. Again, no statistically significant differences were observed (Fig. 3C).

### LysMCre^+/−^/Il-6r^fl/fl^ mice show no difference in susceptibility to acute DSS-induced colitis compared to Il-6r^fl/fl^ control mice

**Figure 4:**
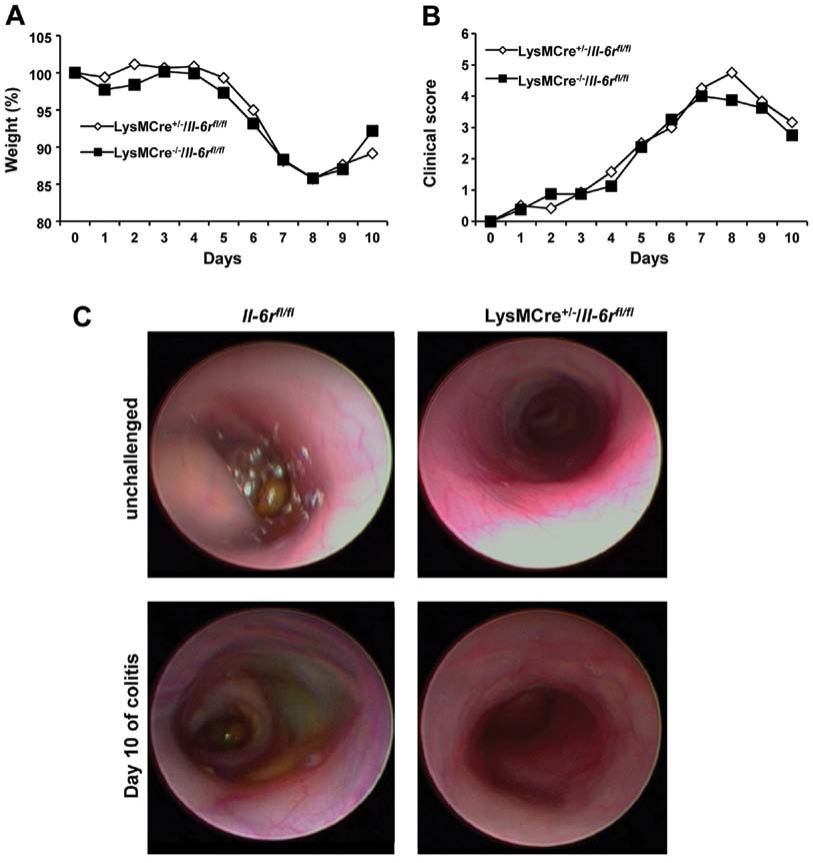
No difference in weight loss, clinical score and endoscopic view was observed in acute dextran sodium sulfate (DSS)-induced colitis between LysMCre^+/−^/*Il-6r^fl/fl^* mice and their littermate controls. (A) Weight curve during acute DSS-induced colitis for LysMCre^+/−^/*Il-6r^fl/fl^* mice and their littermate controls. (B) Clinical score during acute DSS-induced colitis for LysMCre^+/−^/*Il-6r^fl/fl^* mice and their littermate controls. (C) Endoscopic analysis of unchallenged and DSS-treated LysMCre^+/−^/*Il-6r^fl/fl^* mice and their littermate controls. Unchallenged mice were coloscopied and compared to the mice at the end of the experimental period of acute DSS-induced colitis.

We then analyzed the tissue-specific knockout of the *Il-6r* in mice with DSS-induced colitis, in order to exclude the possibility that the global knock out showed no phenotype, as the knock out in the myeloid cell lineage may compensate for the effects of the knock out in other cell types. No significant differences in weight loss or clinical scores between the male LysMCre^+/−^/*Il-6rf^l/fl^* mice and their littermate controls were observed (Fig. 4A and B). In the present sudy, we used 2.5% DSS in drinking water. Therefore, many mice lost too much weight and had to be excluded from the experiment. For the LysMCre^+/−^/*Il-6r^fl/fl^* mice, the mortality rate was 3 out of 9 compared to 7 out of 11 for their littermate controls. This indicates that, under severe conditions of DSS-induced colitis, the knock out of *Il-6r* in neutrophils and monocytes may be beneficial to the survival of mice. When we used 1.5% DSS in the drinking water, this difference was not observed for the first cycle of chronic DSS-induced colitis (Fig. 6), reflecting the situation in a milder form of acute DSS-induced colitis.

We also performed coloscopy, immunohistochemistry, FITC-dextran measurements, ELISA for IL-6 and qPCR for various cytokines and other factors involved in colitis. In brief, we compared the experimental data for the LysMCre^+/−^/*Il-6r^fl/fl^* mice, as well as their littermate controls with the unchallenged mice. As expected from the weight loss curves and clinical scores, we observed no difference in these parameters between the LysMCre^+/−^/*Il-6r^fl/fl^* mice and their littermate controls. We compared the endoscopy of the colon of the challenged and unchallenged LysMCre^+/−^/*Il-6r^fl/fl^* and their littermate controls on day 10 (Fig. 4C). The unchallenged colon shows a normal colonic mucosa. The surface is glistening and smooth, and blood vessels are clearly visible. The challenged colon lost its glistening surface and blood vessels are hard to see or not visible. From a distance, one can see a deep red color, indicating bleeding or inflammation (Fig. 4C).

**Figure 5:**
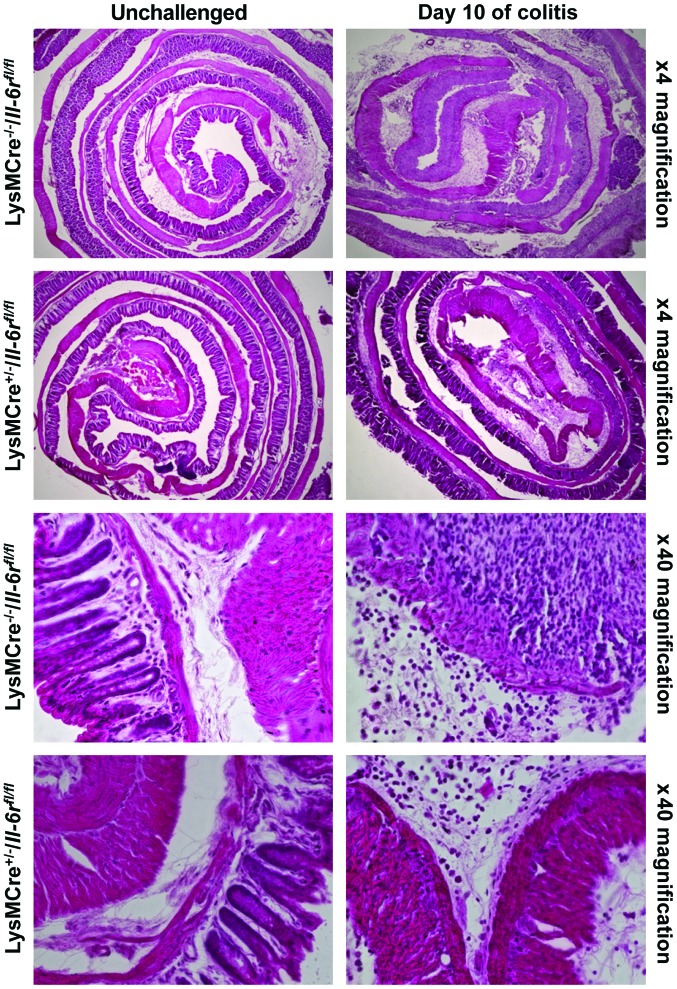
No difference in colon architecture and inflammation was observed between LysMCre^+/−^/*Il-6r^fl/fl^* mice and their littermate controls in acute DSS-induced colitis. Hematoxylin and eosin (H&E) staining of unchallenged and DSS-treated LysMCre^+/−^/*Il-6r^fl/fl^* mice and their littermate controls. Swiss-rolls of the colons of unchallenged mice were stained with H&E and compared to swiss-rolls of the colon of mice at the end of the experimental period of acute DSS-induced colitis. The upper 2 panels show an overview of the complete colon (magnification, ×4), bar of 300 μm; the lower 2 panels show a magnification of the rectum (magnification, ×40), bar of 50 μm. Immunohistochemistry was performed as described in Materials and methods.

Furthermore, we performed hematoxylin and eosin (H&E) staining. The unchallenged mice had normal crypt architecture and no infiltration of immune cells into the mucosa. The DSS-treated mice from day 10 showed a complete loss of rectal crypt structure and a massive infiltration of immune cells (Fig. 5). Again, no differences between the knockout and wt mice were observed.

**Figure 6:**
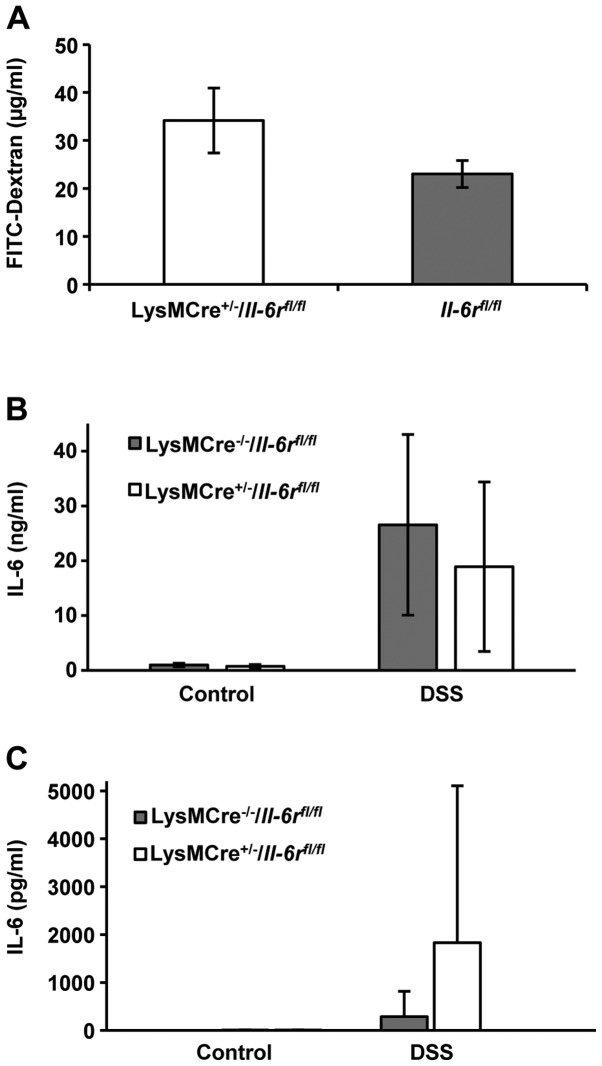
No significant differences were observed in FITC-dextran levels in the serum of LysMCre^+/−^/*Il-6r^fl/fl^* mice and their littermate controls or in the interleukin (IL)-6 level in the serum or colon culture supernatant. (A) FITC-dextran levels in the serum of LysMCre^+/−^/*Il-6r^fl/fl^* mice and their littermate controls at the end of the experimental period of acute DSS-induced colitis. FITC-dextran analysis was performed as described in Materials and methods. Standard errors were calculated and indicated. (B) IL-6 expression level in the cell culture supernatant of the colon culture of LysMCre^+/−^/*Il-6r^fl/fl^* mice and LysMCre^−/−^/*Il-6r^fl/fl^* mice at the end of the experimental period of acute DSS-induced colitis and in unchallenged control mice. Colon culture and IL-6 level determination were performed as described in Materials and methods. Standard errors were calculated and are shown. (C) IL-6 level in the serum of LysMCre^+/−^/*Il-6r^fl/f^*^l^ mice and LysMCre^−/−^/*Il-6r^fl/fl^* mice at the end of the experimental period of acute DSS-induced colitis and in unchallenged control mice. IL-6 level determination was performed as described in Materials and methods. Standard errors were calculated and are shown.

To analyze the permeability of the colon, we administered FITC-dextran by gavage 4 h prior to sacrifice on day 10. FITC-dextran cannot permeate the intact colon epithelium. If the barrier function is compromised, FITC-dextran will enter the bloodstream and can be quantified in mouse serum. We detected higher, although not significantly, FITC-dextran levels in the serum of LysMCre^+/−^/*Il-6r^fl/fl^* mice compared with their littermate controls (Fig. 6A).

On day 10, colon cultures were prepared. No differences in IL-6 secretion from the colon cultures of the LysMCre^+/−^/*Il-6r^fl/fl^* mice and their littermate controls were observed (Fig. 6B). The serum levels of IL-6 were increased in the LysMCre^+/−^/*Il-6r^fl/fl^* mice compared to their littermate controls (Fig. 6C). The difference was not statistically significant.

Finally, using qPCR, we analyzed the expression of *Tgf-β*, *Il-1*, *Tnf-α*, *Kc* and *Vα14* in the LysMCre^+/−^/*Il-6r^fl/fl^* mice and their littermate controls relative to the unchallenged littermate controls (Table I). We found that the expression of the anti-inflammatory cytokine, *Tgf-β*, which is known to play a beneficial role in DSS-induced colitis (24), was slightly upregulated when the mice were challenged with DSS; however, we did not observe a significant difference between the LysMCre^+/−^/*Il-6r^fl/fl^* mice and their littermate controls. The pro-inflammatory cytokine, *Il-1,* was upregulated in both genotypes, and *Tnf-α* and *Kc* expression was not significantly changed. As recently investigated, iNKT cells in the colon can ameliorate DSS-induced colitis (23). Therefore, we screened for differences in the amount of *Vα14*, a marker of iNKT cells, but did not observe any significant differences. Thus, we concluded from these experiments that the course of acute DSS-induced colitis was comparable in the LysMCre^+/−^/*Il-6r^fl/fl^* and control mice.

### LysMCre^+/−^/Il-6r^fl/fl^ mice show no differences in susceptibility to chronic DSS-induced colitis compared to Il-6r^fl/fl^ control mice

**Figure 7:**
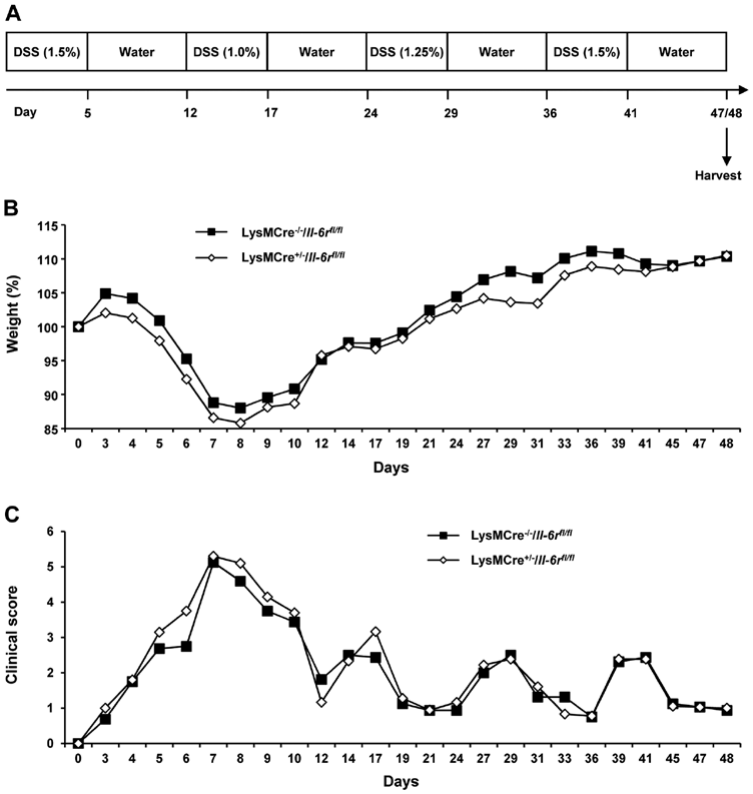
No difference in weight loss and clinical score was observed in chronic DSS-induced colitis between LysMCre+/−/*Il-6rfl/fl* mice and their littermate controls (A) Schematic overview of the experimental procedure of chronic DSS-induced colitis. On day 5, DSS-containing water was removed and replaced by autoclaved tap water for 7 days. On day 12, water was replaced by water containing 1.0% DSS for 5 days, and on day 17 replaced by autoclaved tap water for 7 days, on day 24 replaced by water containing 1.25% DSS for 5 days, and on day 29 replaced by autoclaved tap water for 7 days, on day 36 replaced by water containing 1.5% DSS for 5 days and finally on day 41 replaced by autoclaved tap water for 6 or 7 days as indicated. On day 47 VilCre+/−/*Il-6rfl/fl* mice and their littermate controls, on day 48 LysMCre+/−/*Il-6rfl/fl* mice and their littermate controls were anesthetized and analyzed as described in Materials and methods. (B) Weight curve during chronic DSS-induced colitis for LysMCre+/−/*Il-6rfl/fl* mice and their littermate controls. (C) Clinical score during chronic DSS-induced colitis for LysMCre+/−/*Il-6rfl/fl* mice and their littermate controls.

In order to monitor long-term effects, we created a mouse model of chronic DSS-induced colitis with the LysMCre^+/−^/*Il-6r^fl/fl^* mice and their littermate controls. Briefly, 4 cycles were conducted for 5 days each with DSS in their water followed by 7 days with normal water. DSS concentrations were set to 1.5% for the first cycle, 1.0% for the second cycle, 1.25% for the third cycle and 1.5% for the fourth cycle. The mice were sacrificed after 6 or 7 days with normal water of the final cycle as indicated (Fig. 7A).

We observed no significant differences in weight loss curves and clinical scores between the LysMCre^+/−^/*Il-6r^fl/fl^* mice and their littermate controls (Fig. 7B and C).

## Discussion

IBD affects a great number of individuals in developed and developing countries (2). However, the exact pathophysiological mechanisms underlying the malignancy remain elusive. One important factor is IL-6, which has been shown to be upregulated in colitis (25–28). Even though this suggests that IL-6 plays a negative role in colitis, animal models of colitis induced by DSS (17) have shown contradictory results (11,12). Using female mice and high DSS concentrations in their drinking water, the mortality rate of mice has been shown to be reduced in *Il-6*-deficient mice (12). In contrast to this, male mice deficient in *Il-6* in a DSS-induced colitis using lower concentrations of DSS, developed more severe colitis due to the increased death rate of intestinal epithelial cells (11). One could speculate that the amount of DSS administered is important for the switch in the pro-inflammatory role of IL-6 to its regenerative role. It would also be possible that the gender of the used mice determines the difference in the role of IL-6. In this study, therefore, we decided to use male *Il-6^+/+^* mice, and administer low doses of DSS and to neutralize IL-6 with a neutralizing IL-6 antibody. This was done to mimic the latter experiment (11). Surprisingly, mice deficient in *Il-6* showed significantly reduced weight loss and a lower clinical score than the control animals. Although IL-6 has positive effects on regeneration (16), its role in DSS-induced colitis was shown to be detrimental.

We further showed that although the IL-6R is the only known receptor for IL-6, the LysMCre^+/−^/*Il-6r^fl/fl^* and *Il-6r*^−^*^/^*^−^ mice and their littermate controls had the same susceptibility to DSS-induced colitis. A difference in the mortality rate was observed between the genotypes only in the severe form of DSS-induced colitis.

We proved that the knock out of the *Il-6r* is almost complete in monocytes from LysMCre^+/−^/*Il-6r^fl/fl^* mice. This was shown by flow cytometric analysis of the IL-6R in splenocytes and bone marrow cells. Even though some cells were positively stained for IL-6R and CD11b, this is more likely due to the gating of cells and not to IL-6R expression. Surprisingly, we found that although it has been published that approximately 2/3 of sIL-6R in serum is produced by neutrophils and monocytes (16), the level of sIL-6R in the serum of LysMCre^+/−^/*Il-6r^fl/fl^* was only reduced by approximately 1/3 in the present study. This is likely not due to an incomplete recombination in monocytes, as the flow cytometric data revealed that the recombination was almost perfect. However, we cannot exclude the fact that, for unknown reasons, the recombination was less successful in neutrophils than in monocytes.

We also generated *Il-6r*^−^*^/^*^−^ mice. Our data revealed that the IL-6R does not play any role in DSS-induced colitis. This finding is consistent with data on skin wound healing, where it only plays a minor role compared to IL-6 (16).

There are two possible explanations for the difference observed between *Il-6r*^−^*^/^*^−^ and *Il-6*^−^*^/^*^−^ mice: either the other ligands of IL-6R play a role or IL-6 can also bind to a yet unknown receptor. It is well known that the IL-27 subunit p28 (IL-30) binds to the membrane-bound and sIL-6R, inducing signal transduction via a gp130 homodimer (14,15). However, p28 has been excluded as the important factor contributing to the different results obtained from *Il-6*^−^*^/^*^−^ and *Il-6r*^−^*^/^*^−^ mice in wound healing (16). Another low-affinity ligand for the IL-6R is the widely expressed, CNTF (13,29,30). It reduces the production of pro-inflammatory cytokines (31) and exerts protective effects against experimental autoimmune encephalomyelitis (EAE), a mouse model of multiple sclerosis (32,33). Due to its low affinity, CNTF is unlikely to play a role as the IL-6R ligand that influences the susceptibility to DSS-induced colitis in mice.

At this point of understanding, it would be reasonable to breed double-deficient mice, which express neither IL-6 nor IL-6R (*Il-6*^−^*^/^*^−^*/Il-6r*^−^*^/^*^−^), and challenge them in comparison with *Il-6*^−^*^/^*^−^*/Il-6r^+/+^*, *Il-6^+/+^/Il-6r*^−^*^/^*^−^ and *Il-6^+/+^/Il-6r^+/+^* mice in a model of DSS-induced colitis. CNTF and p28 may play a beneficial role in DSS-induced colitis, as well as another unknown ligand for the IL-6R. The double-deficient mice should then lack the detrimental IL-6 signal and the beneficial alternate IL-6R ligand signal and, therefore, show normal susceptibility to DSS-induced colitis. Alternatively, if the double-deficient mice are as susceptible to DSS-induced colitis as *Il-6*^−^*^/^*^−^*/Il-6r^+/+^* mice, this would suggest that IL-6 has another unknown receptor, which mediates the detrimental IL-6 signal in colitis, while IL-6R plays no role.

## Figures and Tables

**Table I:** Differences in the expression levels of relevant markers between the LysMCre^+/−^/*Il-6r^fl/fl^* mice and their littermate controls.

	LysM^−/−^/*Il-6r^fl/fl^* control	LysM^+/−^/*Il-6r^fl/fl^* control	LysM^−/−^/*Il-6r^fl/fl^* DSS	LysM^+/−^/*Il-6r^fl/fl^* DSS
*Tgf-β*	1±0.40	0.78±0.25	3.40±0.97	2.73±1.65
*Il-1*	1±0.84	1.67±0.34	15.74±14.18	12.80±10.11
*Tnf-α*	1±1.14	2.51±0.43	2.02±0.73	2.42±1.52
Kc	1±0.46	1.25±0.27	0.77±0.17	1.73±0.78
*Vα14*	1±0.43	3.22±1.47	1.32±0.66	1.74±0.35

*Transforming growth factor (Tgf)-β*, *Il-1*, tumor necrosis factor (*Tnf)-α*, *Kc* and *Vα14* mRNA levels in the colons of LysMCre^+/−^/*Il-6r^fl/fl^* mice and LysMCre^−/−^/*Il-6rf^l/fl^* mice at the end of the experimental period of acute DSS-induced colitis and in the unchallenged control mice. qPCR and the harvesting of the colon were carried out as described in Materials and methods. RNA was extracted and reverse-transcribed using oligo(dT) primers. cDNA was used for qPCR in triplicate. *Gapdh* was used as a housekeeping control. The mRNA expression of each marker in the unchallenged LysMCre^−/−^/*Il-6r^fl/fl^* mice was set to mRNA fold 1 and the mRNA expression of the markers in the other groups of mice was calculated relative to the mRNA expression in the unchallenged LysMCre^−/−^/*Il-6r^fl/fl^* mice. Standard errors were calculated and are presented.
